# Computerized tomography scan evaluation after fresh osteochondral allograft transplantation of the knee correlates with clinical outcomes

**DOI:** 10.1007/s00264-022-05373-6

**Published:** 2022-04-12

**Authors:** Pablo Eduardo Gelber, Eduard Ramírez-Bermejo, Alex Grau-Blanes, Aránzazu Gonzalez-Osuna, Oscar Fariñas

**Affiliations:** 1 Department of Orthopaedic Surgery, Hospital de La Santa Creu I Sant Pau, Universitat Autonoma de Barcelona, C/Sant Quintí 89, 08041 Barcelona, Catalunya Spain; 2 ICATME-Hospital Universitari Dexeus, Universitat Autonoma de Barcelona, Barcelona, Spain; 3 Barcelona Tissue Bank, Banc de Sang I Teixits, Barcelona, Spain

**Keywords:** Osteochondral allograft, Cartilage repair, CT, Fresh OCA, Transplantation, Correlation

## Abstract

**Purpose:**

To determine the correlation between the assessment computed tomography osteochondral allograft (ACTOCA) scoring system and clinical outcomes scores. The hypothesis was that the ACTOCA score would show sufficient correlation to support its use in clinical practice.

**Methods:**

We prospectively collected data from all consecutive patients who underwent cartilage restitution with fresh osteochondral allograft (FOCA) transplantation for osteochondral lesions of the knee and had a minimum follow-up of two years. CT scans were performed at three, six and 24 months post-operatively. A musculoskeletal radiologist blinded to the patients’ medical history evaluated the scans using the ACTOCA scoring system. Clinical outcomes collected preoperatively and at three, six and 24 months postoperatively were evaluated using the International Knee Documentation Committee (IKDC), Kujala, the Western Ontario Meniscal Evaluation Tool (WOMET), and the Tegner Activity Scale.

**Results:**

The mean total ACTOCA score showed a statistically significant correlation with the clinical outcome. The correlation was optimal at 24 months. We found a high negative correlation with the IKDC, Kujala and Tegner (− 0.737; − 0.757, and − 0.781 respectively), and a moderate negative correlation with WOMET (− 0.566) (*p* < 0.001). IKDC, Kujala, WOMET, and Tegner scores showed a significant continuous improvement in all scores (*p* < 0.001).

**Conclusion:**

The mean total ACTOCA score showed a linear correlation with clinical results in IKDC, Kujala, WOMET, and Tegner scores, being the highest at 24 months post-surgery. This finding supports the use of ACTOCA to standardize CT scan reports following fresh osteochondral allograft transplantation in the knee.

## Introduction

Osteochondral knee lesions in active young patients have a devastating effect on daily life [1]. Large symptomatic osteochondral lesions are a complex treatment challenge [2]. If untreated, progressive worsening of tibiofemoral osteochondral lesions and evolution to osteoarthritis can be expected [3]. In patellofemoral osteochondral lesions, an evolution to osteoarthritis has not been described [4], but surgery of symptomatic osteochondral lesions in the patellofemoral joint have to be considered when non-operative treatment fails [5].

Osteochondral lesions larger than 2 cm^2^ are the main indication for FOCA transplantation where osteochondral cores from a size-matched, fresh cadaver are matched to the patient’s knee injury [6]. Good clinical and functional outcomes can be expected after FOCA transplantation, even at longer follow-up [7–10].

The imaging assessment of bone aspects such as cystic changes and osseous integration is key to graft survival after FOCA transplantation [2]. As strong evidence is lacking as to whether magnetic resonance imaging (MRI) is reliable to correlate with clinical outcome scores [11, 12], a semiquantitative ACTOCA scoring system was recently developed and validated [13]. The ACTOCA includes five CT features relative to the aspect of the transplanted graft and the host bone (graft signal density, osseous integration, surface percentage with a discernible cleft, cystic changes, and presence of intra-articular fragments). However, the correlation between ACTOCA scores and clinical outcome scores has not yet been explored.

The objective of this study was to determine the correlation between ACTOCA scores and clinical outcome scores. The hypothesis was that the ACTOCA score would show sufficient correlation to support its use in clinical practice.

## Material and methods

In this prospective study, we included all consecutive patients undergoing cartilage repair with FOCA transplantation for osteochondral knee lesions between August 2017 and August 2019. Surgery was carried out by a single surgeon at an academic medical centre, and all patients had a minimum follow-up of two years.

Inclusion criteria were patients younger than 50 years undergoing cartilage repair with FOCA transplantation for symptomatic osteochondral knee lesions with chronic onset after a minimum of six months of non-operative treatment in accordance with standard clinical practise at our institution. The surgical procedure was indicated in patients with large focal full-thickness chondral and osteochondral defects (> 2 cm^2^) on the tibial plateau, femoral condyles, trochlea, and/or patella.

Concomitant realignment osteotomy was performed in cases of tibiofemoral FOCA with tibiofemoral malalignment greater than 3° from the neutral mechanical axis into the involved compartment. Patellofemoral joints with a TTTG distance greater than 15 mm had an associated tibial tubercle anteromedialization osteotomy. Concomitant meniscal insufficiency was corrected with lateral or medial meniscal allograft transplantation, as needed. Exclusion criteria were inflammatory arthritis, large degenerative lesions comprising all three compartments, BMI > 30 kg/m^2^, diabetes, systemic inflammatory diseases, infection or history of osteomyelitis in the graft recipient area, and active neoplasia.

The study was approved by the ethics committee of our institution (IIBSP-ALO-2018–21). Informed consent was obtained from each patient following the guidelines laid down by our local ethics committee.

### Surgical technique

An arthroscopic evaluation of all compartments of the knee was performed to confirm the size and depth of the lesion and to address any concurrent intra-articular pathology.

Any anatomic deformity or biomechanical alteration of the tibiofemoral joint and/or patellofemoral joint was corrected to avoid further cartilage degradation of the graft.

The articular cartilage defect was sized and reamed to a depth of approximately 8 to 10 mm. Fresh osteochondral allografts were obtained following screening and processing requirements of the local authorized tissue bank. The osteochondral allograft was irrigated using pulsatile lavage. A bone-dowel technique was performed for isolated defects with a well-defined affected area in an easily accessible surface of the knee such as the femoral condyles, mid-patella, or trochlea. The shell technique was used for asymmetric lesions, such as those involving the whole patella or those affecting a high-degree dysplastic trochlea. In cases of posttraumatic complex lesions of the tibial plateau with a concomitant meniscal deficiency, we transplanted a 10-mm-high medial or lateral tibial plateau including the corresponding meniscus. The bone-dowel technique obtained a press-fit fixation. Other techniques required fixation with bioabsorbable pins or interfragmentary screws [14–16].

In the first phase of rehabilitation, from zero to six weeks, the goal was graft protection by avoidance of weight-bearing. The day after surgery, progressive range of motion (ROM) exercises using a continuous passive motion device were started. Weight-bearing and ROM varied based on several variables but the goal was to avoid stressing the transplanted graft. A gradual transition to partial and full weight-bearing was allowed after six to ten weeks. [9]

### CT assessment

CT scans were performed postoperatively on day one to rule out any technical errors and then at three, six and 24 months. Post-operative CT studies were obtained on a 16-multidetector system (Brillance, Philips Healthcare) using a reduced dose protocol with the lowest scan length required to include the allograft. Multiplanar reformatted 2-mm contiguous sagittal and coronal images were later obtained. Collimation was performed for all CTs to increase image quality and reduce the patient’s overall radiation exposure.

**Fig. 1:**
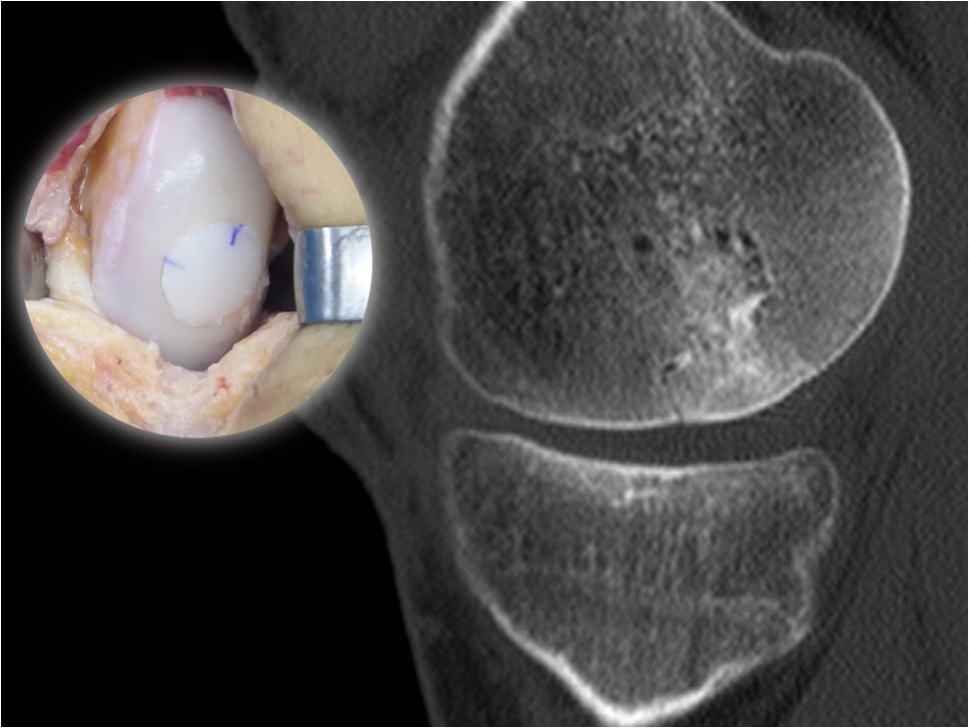
CT scan taken at 6 months and surgical image of a medial femoral condyle FOCA obtaining a low ACTOCA score (1 point)

**Fig. 2:**
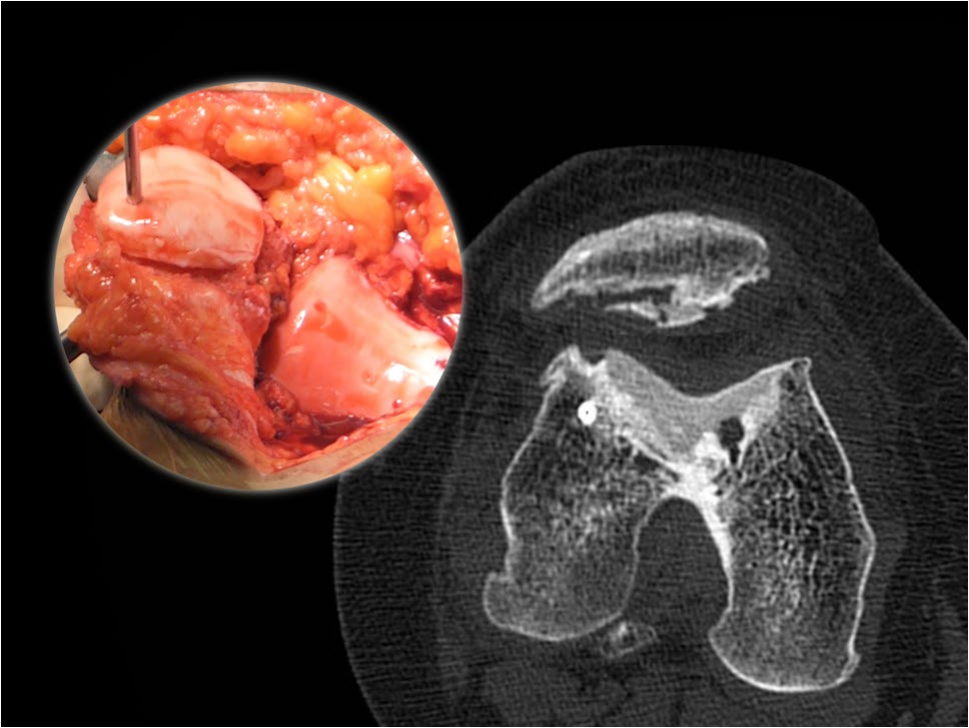
CT scan taken at 24 months and surgical image of a trochlear and patellar FOCA obtaining a high ACTOCA score (6 points)

For this imaging study, we used the previously published and validated comprehensive ACTOCA score [13]. The ACTOCA includes five CT features relative to the aspect of the transplanted graft and the host bone; graft signal density, osseous integration, surface percentage with a discernible cleft, cystic changes, and presence of intra-articular fragments. Axial views were used to evaluate the patella-femoral joint, and sagittal views were used to evaluate the femoral condyles and tibia. Each parameter was scored, and the total summation was calculated. A lower total score indicates better incorporation of the graft, with possible scores ranging from zero to eight (Table 1, Figs. 1 and 2).

**Table 1:** ACTOCA scoring system

CT features	CT score
1. Graft signal density relative to host bone	0: Equivalent
	1: Superior
	2: Inferior
2. Osseous integration at host-graft junction	0: Crossing trabeculae
	1: Discernible cleft < 3 mm
	2: Discernible cleft > 3 mm
3. Surface percentage with a discernible cleft at host-graft junction	0: < 30%
	1: > 30%
4. Cystic changes of graft and/or host-graft junction	0: Absent
	1: Present < 3 mm
	2: Present > 3 mm
5. Presence of intraarticular fragments	0: Absent
	1: Present

All CT scans were evaluated by a musculoskeletal radiologist blinded to the patient’s medical history.

### Functional evaluation

Clinical results were collected preoperatively and at three, six and 24 months post-operatively.

At each time point, participants completed several patient-reported outcome instruments to measure clinical results. The scores used were the IKDC, Kujala, WOMET, and the Tegner activity scale [17–20].

### Secondary outcomes

Sociodemographic data were collected at baseline to characterize the study sample and explore age, sex at birth, involved side, and BMI as potential confounding variables. Concomitant procedures (osteotomy, ligamentous repair/reconstruction, meniscal allograft transplantation) were recorded at the time of surgery. Osteochondral allograft type (patellofemoral, femoral condyle, or tibial) was also noted.

### Statistical analysis

Statistical analysis was performed using the statistical package IBM SPSS V26.0 (IBM Corp. Armonk, NY). Descriptive statistics were used to determine patient and lesion characteristics. The results are given as a number of cases and/or percentage for categorical data, and as mean, standard deviation and range for quantitative data. Variables repeated during the trial (functional scales and CT) were analysed using ANOVA tests for repeated measures with Greenhouse–Geisser correction to avoid sphericity. The correlation between clinical results and imaging results was analysed by Pearson’s correlation coefficient. The overall level of significance was set at 0.05 for two-sided tests.

The power calculation was done according to IKDC from preoperative to 24 months postoperatively. A 5-point threshold for clinical relevance was set a priori. This number is in fact lower than multiple reported studies to detect minimal changes and similar to what was reported in a recent study by Magnuson et al. [21]. According to the power calculation, to generate a power of 80%, an alpha of 0.05, and a standard deviation of 10 points, this study required 30 patients.

## Results

A total of 38 patients (24 males; 63%) met the inclusion criteria. The mean post-operative follow-up was 38 months (range, 30–48 months). Patients’ mean age was 36.63 ± 6.63 years (range, 18–46 years). Thirty-one of the 38 patients (81.6%) received unipolar OCA transplants, defined as involving ≥ one non-apposing articulating surfaces, and 71 (18.4%) received bipolar transplants, defined as involving two opposing articulating surfaces. Baseline demographic data and clinical characteristics are presented in Table 2.

**Table 2:** Patient demographics and specific knee data (*N* = 38)

Factor		
Age (years)	36.63 ± 6.63 23.92 ± 2.57 24/14	
BMI		
Male/female		
Lesion location		
	Femoropatellar joint (%) -Isolated patella (%) -Femoral groove + patella (%)	55.3 76 24
	Femoral condyle (%) -Medial (%) -Lateral (%)	34.2 70 30
	Tibia (%) -Medial (%) -Lateral (%)	10.5 75 25
FOCA type		
	Unipolar (%)	81.6
	Bipolar (%)	18.4
FOCA technique		
	Plug (%)	55.3
	Shell (%)	34.2
	Small fragment (%)	10.5
Tibial tubercle osteotomy (%)	18.4	
High tibial osteotomy (%)	18.4	

No statistically significant differences were noted for ACTOCA or functional scales (IKDC, Kujala, WOMET, or Tegner) according to sex at birth, age, BMI, concomitant procedures, or osteochondral allograft type. Regarding osteotomies, no statistically significant differences were found between patients with or without osteotomies on CT evolution (*p* = 0.819), IKDC evolution (*p* = 0.139), Kujala evolution (*p* = 0.158), WOMET evolution (*p* = 0.299), and Tegner evolution (*p* = 0.138).

### Evolution of clinical scores

Pre-operative and post-operative comparisons of clinical scores at three, six and 24 months showed a significant continuous improvement in IKDC, Kujala, WOMET, and Tegner scores (*p* < 0.001) (Table 3).

**Table 3:** Clinical scores

	Preop	3 months	6 months	24 months	Greenhouse–Geisser
IKDC	31.26 ± 9.4 (15–53)	41 ± 10.95 (21–65)	47.58 ± 13.5 (20–76)	60.47 ± 18.81 (20–88)	< 0.001
Kujala	38.84 ± 12.46 (17–63)	49.63 ± 12.87 (27–76)	58.13 ± 14.4 (30–94)	69.5 ± 17.1 (30–97)	< 0.001
WOMET	38.74 ± 14.87 (13–79)	46.68 ± 15.07 (18–75)	53.13 ± 16.48 (14 – 87)	65.5 ± 18.2 (25–98)	< 0.001
Tegner	1.97 ± 0.91 (1–4)	1.89 ± 0.89 (1–4)	2.08 ± 0.78 (1–4)	2.76 ± 1.03 (1–4)	< 0.001

### ACTOCA evolution

The ACTOCA scores improved significantly at three, six and 24 months post-surgery (*p* < 0.001) (Table 4).

**Table 4:** ACTOCA scores

	3 months	6 months	24 months	Greenhouse–Geisser (*p*)
ACTOCA	2.16 ± 0.92 (0–4)	1.34 ± 1.21 (0–4)	1.05 ± 1.33 (0–4)	< 0.001

The values are given as the mean ± standard deviation with the range in parentheses

### Correlation between clinical outcomes and mean ACTOCA score

The total ACTOCA score correlated with the clinical results (Table 5).

**Table 5:** Correlation between total ACTOCA score and clinical outcomes scores

		Pearson correlation coefficient	*p* value
IKDC	3 months	− 0.116	0.488
	6 months	− 0.535	0.001*
	24 months	− 0.737	< 0.001*
KUJALA	3 months	− 0.027	0.872
	6 months	− 0.343	0.035*
	24 months	− 0.757	< 0.001*
WOMET	3 months	− 0.069	0.682
	6 months	− 0.274	0.096
	24 months	− 0.566	< 0.001*
TEGNER	3 months	− 0.177	0.287
	6 months	− 0.313	0.056
	24 months	− 0.781	< 0.001*

^*^Significant

We observed a moderate negative correlation with the IKDC score at six months (Pearson correlation coefficient, − 0.535; *p* = 0.001) and a high negative correlation with IKDC at 24 months (Pearson correlation coefficient, − 0.737; *p* < 0.001). There was a low negative correlation with the Kujala score at six months (Pearson correlation coefficient, − 0.343; *p* = 0.035) and a high negative correlation with Kujala at 24 months (Pearson correlation coefficient, − 0.757; *p* < 0.001). The correlation with WOMET at 24 months showed a low negative correlation (Pearson correlation coefficient, − 0.566; *p* < 0.001), and the correlation with Tegner at 24 months showed a high negative correlation (Pearson correlation coefficient, − 0.781; *p* < 0.001).

## Discussion

The main finding of this study was that the ACTOCA score showed a statistically significant correlation with the clinical outcome. This correlation between the mean total ACTOCA score and the clinical outcome was the highest at 24 months after surgery. At this time, IKDC, Kujala, and Tegner showed a high negative correlation with the ACTOCA score and a moderate negative correlation with WOMET.

To our knowledge, this is the first study to analyse the correlation between CT and clinical outcomes using ACTOCA scores. To date, the gold standard imaging modality to assess graft incorporation after fresh osteochondral allograft has been MRI. However, recent studies have shown that the MRI total score does not correlate meaningfully with clinical outcome scores. In a systematic review and meta-analysis of 32 studies carried out to evaluate the correlation between clinical outcome and MRI after cartilage repair, Windt et al. [22] found conclusive evidence that such correlation was lacking. In another study, Wang et al. [12] investigated 43 patients treated with FOCA after a previous cartilage repair surgical procedure. They found that the total OCAMRISS score, one of the most widely used MRI scores, did not correlate meaningfully with clinical outcome scores. Other authors have also failed to find a correlation between MRI scores and clinical results [23, 24]. In contrast with these results using MRI, using the ACTOCA scoring system, we found a high correlation between CT scan and clinical results. This difference may be due to CT scans offering a better evaluation of bone integration and cystic changes that have been shown to have a great impact on clinical results after FOCA.

The recently developed and validated ACTOCA scoring system [13] includes five CT features: density relative to host bone, integration at the host-graft junction, surface percentage with a discernible cleft at the host-graft junction, cystic changes, and intra-articular fragments. Interobserver agreement was found to be was moderate to substantial for all CT score components, and intra-observer agreement was moderate to almost perfect for all CT score components (*κ* > 0.5, *p* < 0.05), showing that ACTOCA score is a reliable scoring system to evaluate osteochondral allograft transplants.

Although imaging assessment of bone aspects such as osseous integration and cystic changes is of great importance to graft survival after FOCA transplantation, few studies have evaluated this transplantation using CT. Anderson et al. [25] recently developed a CT scoring system and evaluated the relationship of OCA bone parameters measured on CT with clinical outcomes. However, unlike our study, only one postoperative CT scan was collected (at a mean of 5.8 months after surgery), and the clinical score the closest to CT findings was used. This score, therefore, reflected a different post-surgery period for each patient, and this could have made their results less conclusive.

Brown et al. [26] investigated osseous integration and early clinical results following FOCA with cylindrical grafts to the femoral condyle. They reported an overall CT assessment of graft incorporation as a percentage of incorporation based on CT images and found the mean level of incorporation of all grafts was grade 2 (50–75%). They did not, however, evaluate the correlation between clinical outcomes and the percentage of incorporation on CT. Cook et al. [27] reported their results of a series of 18 patients who underwent OATS to the femoral condyle, evaluating CT arthrograms post-operatively. Similarly to other imaging studies of FOCA procedures, again CT arthrograms did not correlate with functional outcomes. It may be because they only evaluated bony integration and articular congruity.

In our study, using the ACTOCA scoring system to evaluate FOCA from CT images, we found a statistically significant correlation with clinical outcomes. Furthermore, the pre-operative and post-operative clinical scores at three, six and 24 months reflected a significant, continuous improvement on IKDC, Kujala, WOMET, and Tegner scores.

The present study has several limitations. First, there was no comparison group, and the sample size was small. In addition, the cohort was relatively heterogeneous with respect to osteochondral allograft type and concomitant procedures. However, no statistically significant differences were noted for ACTOCA or functional scales (IKDC, Kujala, WOMET, or Tegner) according to sex at birth, age, BMI, concomitant procedures, or osteochondral allograft type.

The absence of differences between patients with or without osteotomy may be related to the fact that osteotomies were performed only in cases of tibiofemoral FOCA with tibiofemoral malalignment greater than 3° from the neutral mechanical axis into the involved compartment or in case of patellofemoral FOCA with TTTG distance greater than 15 mm. The remaining cases had normal preoperative values. Therefore, patients with osteotomy and without osteotomy presented a comparable alignment once operated.

Second, all CT scans were evaluated by a single musculoskeletal radiologist blinded to the patient’s medical history. Nevertheless, a recent study showed that ACTOCA provides a moderate to a substantial interobserver agreement and a moderate-to-almost-perfect intra-observer agreement [13]. And third, CT scans expose patients to high doses of radiation. This limitation, however, was significantly reduced with the optimal collimation protocol used.

## Conclusions

The mean total ACTOCA score showed a linear correlation with clinical results in IKDC, Kujala, WOMET, and Tegner scores, being the highest at 24 months post-surgery. This finding supports the use of ACTOCA to standardize CT scan reports following fresh osteochondral allograft transplantation in the knee.
